# Supplementary material for: Mutation-derived, genomic instability-associated lncRNAs are prognostic markers in gliomas
Source: PeerJ. 2023 Aug 2;11:e15810. doi: 10.7717/peerj.15810 (PMC10404032; doi:10.7717/peerj.15810)
Supplement: Supplemental Information 1 [file peerj-11-15810-s001.pdf]

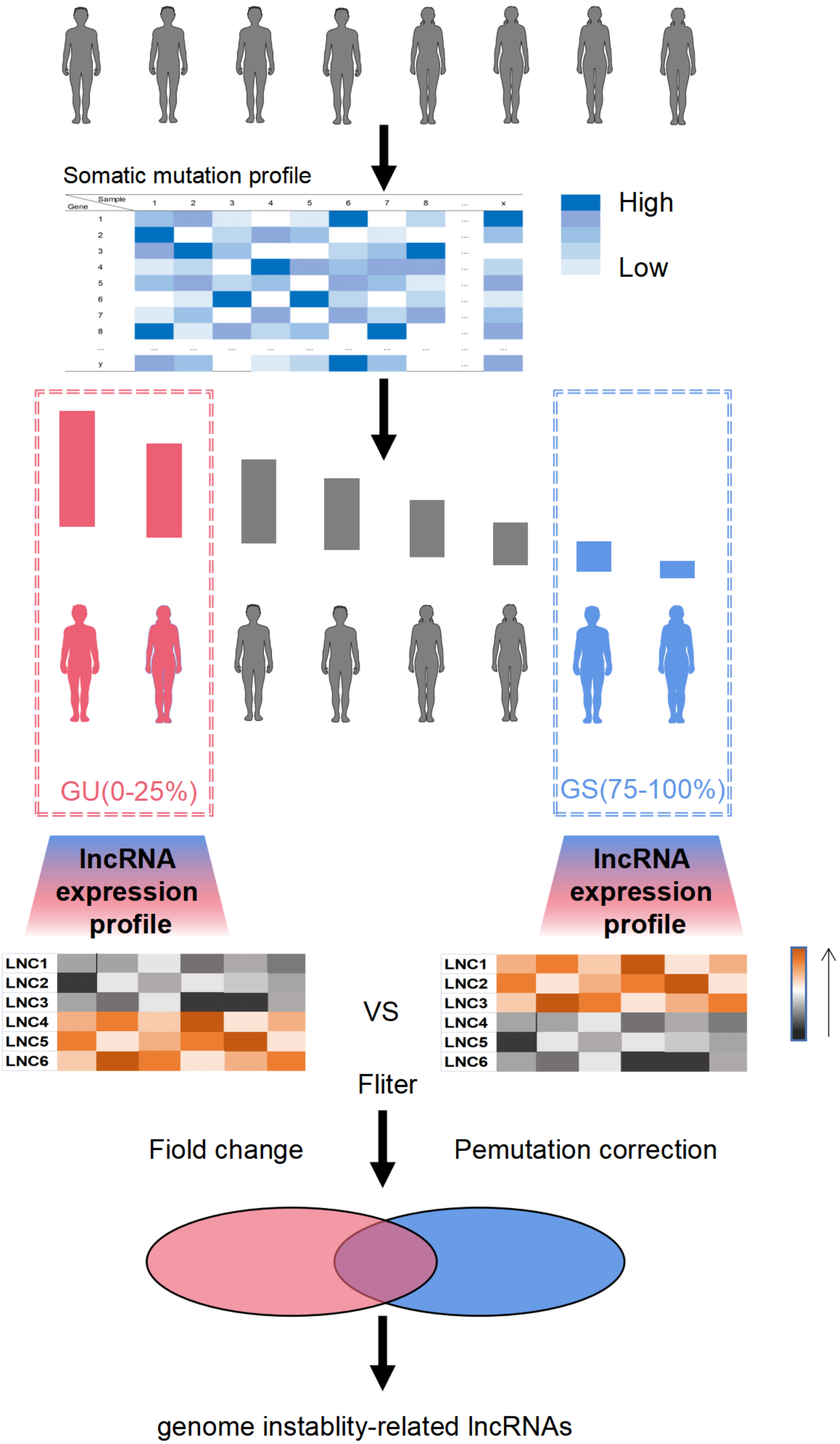

Somatic mutation profile
Sample
Gene
High
Low
GU(0-25%)
GS(75-100%)
lncRNA expression profile
lncRNA expression profile
LNC1
LNC2
LNC3
LNC4
LNC5
LNC6
VS
Fliter
Fiold change
Pemutation correction
genome instablity-related lncRNAs
