# Supplementary material for: Mutation-derived, genomic instability-associated lncRNAs are prognostic markers in gliomas
Source: PeerJ. 2023 Aug 2;11:e15810. doi: 10.7717/peerj.15810 (PMC10404032; doi:10.7717/peerj.15810)

	pvalue	Hazard ratio
LINC01579	<0.001	1.210(1.160−1.262)
AL022344.1	<0.001	0.125(0.069−0.229)
HOXC−AS1	<0.001	1.258(1.165−1.359)
AC025171.5	<0.001	1.982(1.724−2.279)
AC007344.1	<0.001	1.274(1.160−1.399)
MIR4435−2HG	<0.001	1.250(1.192−1.310)
LINC00836	<0.001	0.788(0.728−0.853)
LINC01116	<0.001	1.123(1.090−1.157)
LINC02525	<0.001	1.851(1.566−2.187)
MIR155HG	<0.001	1.721(1.556−1.904)
PIK3CD−AS2	<0.001	1.475(1.345−1.617)
AC005532.1	<0.001	1.088(1.035−1.143)
EGFR−AS1	<0.001	1.749(1.365−2.242)
AC131097.3	<0.001	2.225(1.900−2.606)
LINC00906	<0.001	0.288(0.177−0.470)
AC092957.1	<0.001	1.179(1.093−1.272)
AC016168.2	<0.001	1.321(1.154−1.513)
CYTOR	<0.001	1.280(1.228−1.334)
FAM201A	<0.001	0.192(0.108−0.343)
BX470102.1	<0.001	2.380(1.948−2.907)
MIR924HG	<0.001	1.970(1.661−2.337)
AC015540.1	<0.001	0.555(0.477−0.646)
AL390755.1	<0.001	1.013(1.009−1.017)
SLC25A21−AS1	<0.001	0.383(0.314−0.467)
LHX5−AS1	<0.001	0.795(0.721−0.876)
LINC02636	<0.001	0.077(0.037−0.161)
ELDR	<0.001	1.175(1.093−1.263)
HAR1A	<0.001	0.475(0.365−0.618)
OBI1−AS1	<0.001	0.724(0.608−0.863)
CNIH3−AS2	<0.001	1.225(1.155−1.298)
AL035446.1	<0.001	1.047(1.034−1.061)
MADD−AS1	<0.001	0.073(0.030−0.180)
DNMBP−AS1	<0.001	0.565(0.408−0.782)
H19	<0.001	1.010(1.007−1.013)
AL049871.1	<0.001	1.174(1.085−1.269)
AC008080.4	<0.001	1.138(1.088−1.191)
AL133415.1	<0.001	1.311(1.252−1.373)
AC010655.2	<0.001	1.592(1.408−1.800)
SOCS2−AS1	<0.001	1.099(1.065−1.134)
HOTAIR	<0.001	1.165(1.107−1.225)
SNHG18	<0.001	1.332(1.261−1.408)
AC021242.3	<0.001	1.145(1.083−1.210)
LINC01831	<0.001	1.513(1.359−1.685)
AC022239.1	<0.001	0.252(0.120−0.531)
CNIH3−AS1	0.004	1.143(1.045−1.251)
AL022313.4	<0.001	0.885(0.852−0.918)
AC091932.1	<0.001	1.243(1.144−1.350)
AL391845.2	0.006	0.663(0.496−0.887)
AL512785.1	<0.001	1.090(1.048−1.134)
AGAP2−AS1	<0.001	1.011(1.006−1.016)
AC092958.1	<0.001	1.073(1.043−1.104)
FOXD3−AS1	<0.001	1.136(1.100−1.174)
CDK6−AS1	<0.001	1.137(1.098−1.177)
AC002428.2	<0.001	0.627(0.480−0.820)
AC114803.1	<0.001	1.297(1.183−1.422)
LINC02593	<0.001	0.499(0.410−0.606)
AL139246.1	<0.001	0.172(0.081−0.365)
AL354863.1	<0.001	0.325(0.190−0.555)
AC110015.1	<0.001	1.273(1.152−1.407)
AL596087.2	<0.001	1.151(1.103−1.202)
LINC01956	<0.001	1.443(1.314−1.584)
HOXB−AS1	<0.001	1.259(1.172−1.353)
AC074351.1	<0.001	1.070(1.041−1.100)
LINC02058	<0.001	0.620(0.509−0.756)
AL121827.2	<0.001	0.958(0.945−0.972)
AL354919.2	<0.001	1.068(1.049−1.088)
AC109439.1	<0.001	0.552(0.450−0.678)
AL390786.1	<0.001	0.219(0.117−0.410)
HAR1B	<0.001	0.285(0.176−0.460)
LINC02308	<0.001	1.181(1.118−1.248)
AC064875.1	<0.001	1.265(1.199−1.334)
HOTAIRM1	<0.001	1.029(1.017−1.041)
LINC02587	<0.001	1.047(1.033−1.061)
AC023421.1	<0.001	0.380(0.244−0.591)
AL355916.2	<0.001	0.920(0.888−0.953)
AC062021.1	<0.001	0.881(0.843−0.922)
AC125616.1	0.002	0.599(0.431−0.832)
AC097713.1	<0.001	1.141(1.074−1.212)
CRNDE	<0.001	1.239(1.189−1.292)
AC026790.2	<0.001	0.328(0.201−0.535)

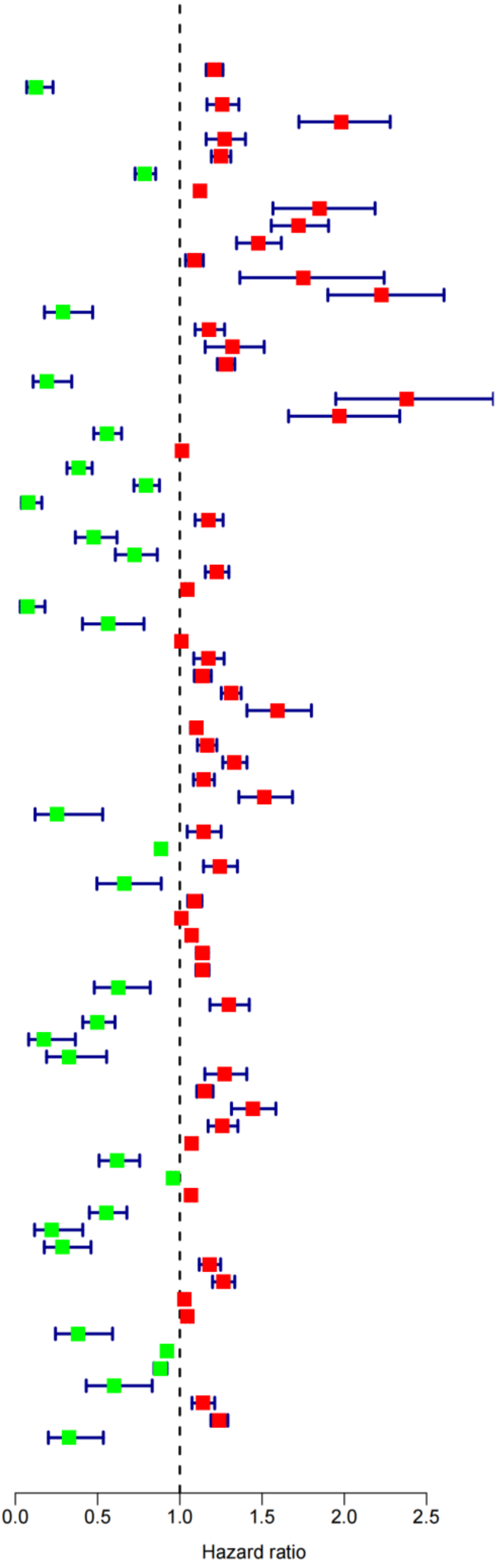

Supplement: Supplemental Information 2 [file peerj-11-15810-s002.pdf]
