# Supplementary figures and images for: Mutation-derived, genomic instability-associated lncRNAs are prognostic markers in gliomas
Source: PeerJ. 2023 Aug 2;11:e15810. doi: 10.7717/peerj.15810 (PMC10404032; doi:10.7717/peerj.15810)

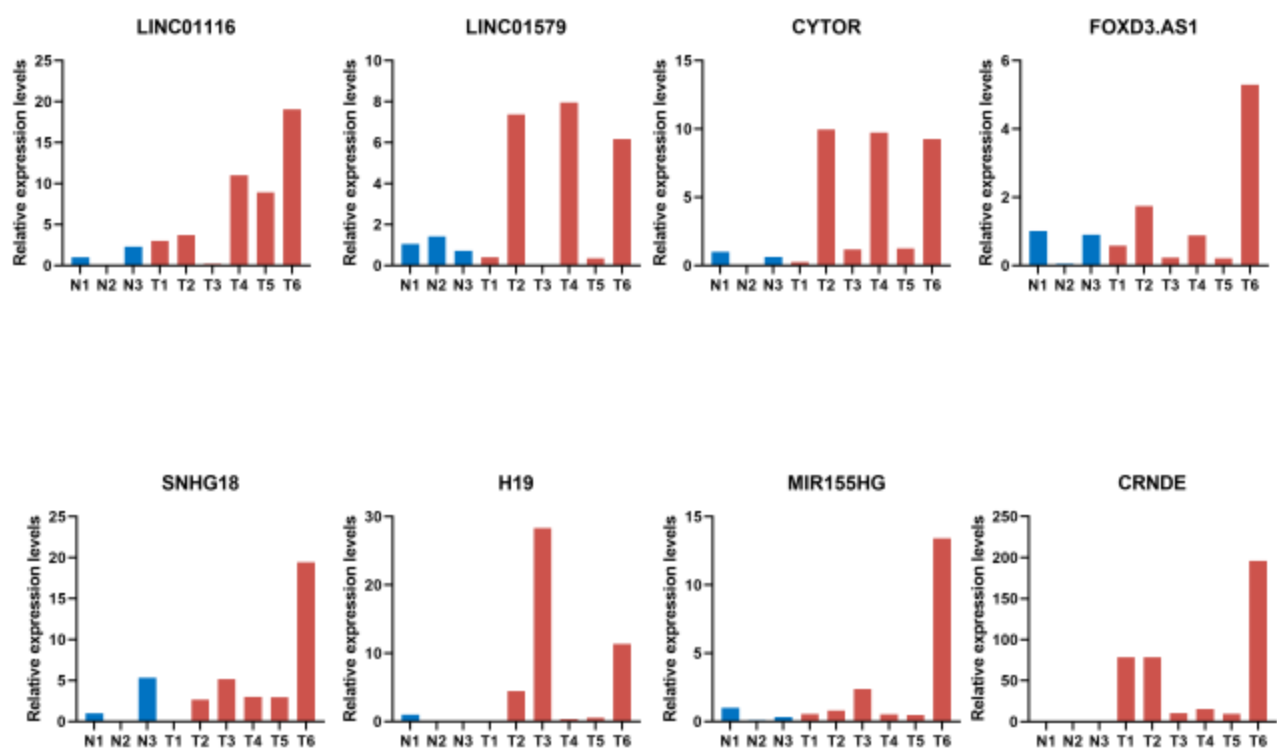

Supplement: Supplemental Information 4 [file peerj-11-15810-s004.pdf]
