# Supplementary material for: Mutation-derived, genomic instability-associated lncRNAs are prognostic markers in gliomas
Source: PeerJ. 2023 Aug 2;11:e15810. doi: 10.7717/peerj.15810 (PMC10404032; doi:10.7717/peerj.15810)
Supplement: Supplemental Information 3 [file peerj-11-15810-s003.pdf]

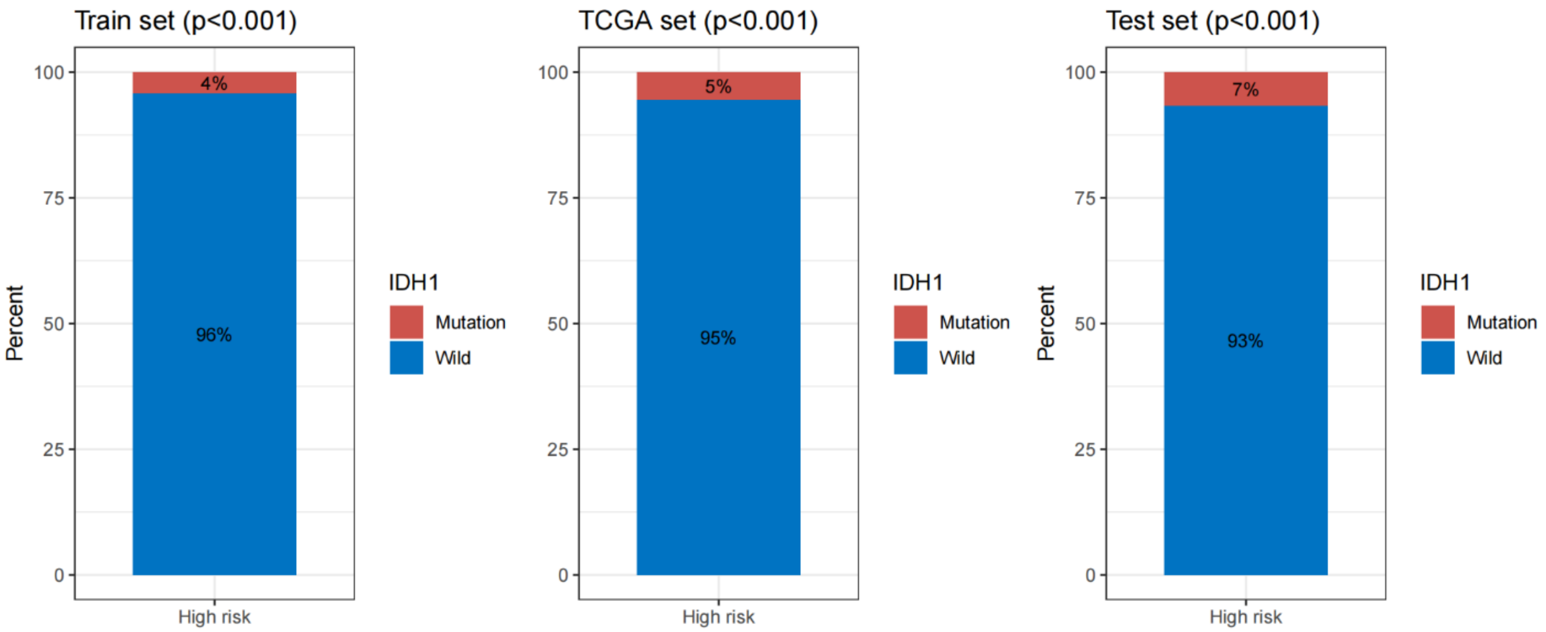
Train set (p<0.001)
TCGA set (p<0.001)
Test set (p<0.001)
Percent
100
75
50
25
0
4%
96%
5%
95%
7%
93%
High risk
IDH1
Mutation
Wild

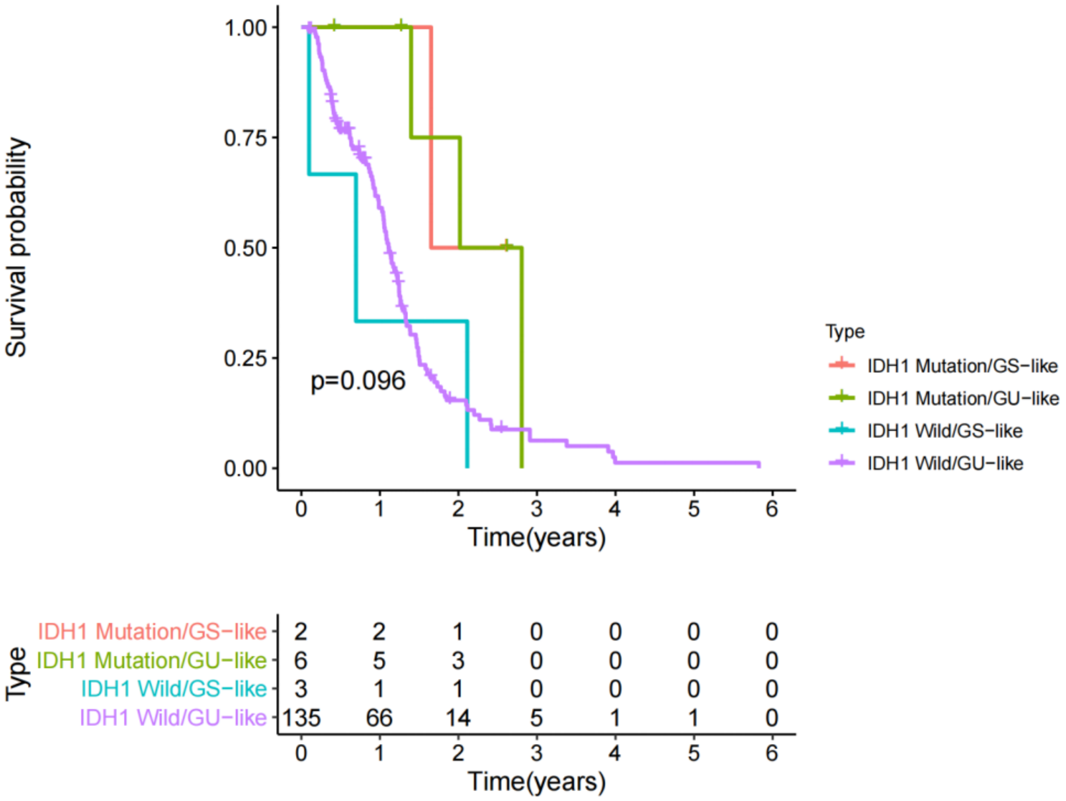
Survival probability
1.00
0.75
0.50
0.25
0.00
p=0.096
Time(years)
0 1 2 3 4 5 6
Type
IDH1 Mutation/GS−like
IDH1 Mutation/GU−like
IDH1 Wild/GS−like
IDH1 Wild/GU−like
Type
IDH1 Mutation/GS−like 2 2 1 0 0 0 0
IDH1 Mutation/GU−like 6 5 3 0 0 0 0
IDH1 Wild/GS−like 3 1 1 0 0 0 0
IDH1 Wild/GU−like 135 66 14 5 1 1 0
0 1 2 3 4 5 6
Time(years)
